# Introduction to the Special Issue on Recent Advances in Mathematics Education

**DOI:** 10.3390/ejihpe12100103

**Published:** 2022-10-04

**Authors:** Michael Gr. Voskoglou, Joanna Mamona-Downs

**Affiliations:** 1 Department of Applied Mathematics, Faculty of Engineering, University of Peloponnese, 26334 Patras, Greece; 2 Department of Mathematics, Faculty of Positive Sciences, University of Patras, 26500 Patras, Greece

The Special Issue with the title “Recent Advances in Mathematics Education” presents contemporary research outcomes regarding the teaching and learning of Mathematics at all levels of Education. It concentrates on themes currently examined in Mathematics Education research, like Tasks’ design, Multiple Solution Tasks, Relating Mathematical Knowledge and Cognitive Variables, scrutinizing teachers’ didactical approaches to enhance students’ Metacognitive Skills, etc. The subjects of the presented fieldwork, (where one appears), range from the fourth to eight grade students as well as teacher students. They were 5 articles accepted for publication among the 10 in total manuscripts submitted to the Special Issue. These articles were published in Volumes 11 (2021) and 12 (2022) of the MDPI *“European Journal of Investigation in Health Psychology and Education”* and include:

- A framework for designing assessment tasks in Mathematics classrooms
- A proposition for understanding algebraic computations from formulas to functions through Geometry
- A theoretical study on the language of the “Rate of Change” in Mathematics
- A study on the personal need for structure and fractions in Mathematics Education, and
- A research on Mathematics teachers’ encouragement of their students’ metacognitive processes

More explicitly, in [1] *E*. *Demosthenous, C. Christou*, and *D. Pitta-Pantazi* (University of Cyprus), develop a framework providing a lens to capture the interplay between the design of mathematics assessment tasks and the analysis of students’ responses. The proposed framework consists of three types of mathematics assessment tasks, their respective competencies, and the characterization of students’ responses. The framework is exemplified with students’ responses from a fourth-grade classroom, and is used to sketch different students’ profiles.

In [2], *A. Barana* (University of Turin, Italy), proposes a set of interactive activities for eighth grade students, with a functional approach to formulas in a geometric context. The goal of the study is to investigate how similar activities can help students to develop multiple approaches to problems, understand algebraic formulas, and discern which main challenges they face. The activities are tested with about 300 students, and the data are analyzed to answer the research questions.

In [3], *E. Avgerinos* and *D. Remoundou* (University of Aegean, Greece), study the language used in mathematics, focusing on the word change by examining the “rate of change” and students’ misconceptions on the issue. Language is an essential aspect of teaching and learning mathematics, being necessary for communication, for the transmission of concepts and ideas, and the formation of the meaning of mathematical concepts.

The research undertaken by *V. Svecova*, *L. Rybansky* and *G. Pavlicova* (Constantine the Philosopher University, Slovakia), which is described in [4], is focused on finding relations between mathematical knowledge and cognitive individual variables. An experiment was realized with 162 students of the Constantine the Philosopher University in Nitra, Slovakia. The relationships between the factors of the personal need for structure (PNS) scale and the knowledge of fractions were determined by the IRT model. A negative correlation was found between the successful solving of fraction test and score in the PNS scale, which means that the higher the success rate of solving the fraction tasks, the lower the overall score on the personal need for structure scale and its subfactors.

In the last paper [5] *W. Daher* and *I. Hashash* (An-Najah National University, Palestine) conducted a research on teachers’ practices to encourage their students’ metacognitive skills. They presented the results of the validity and reliability of a questionnaire that measured teachers’ encouragement of students’ planning, monitoring, regulating, and evaluating. They also utilized statistical tests to investigate the differences in teachers’ metacognitive performing due to gender, to academic qualifications and to the years of experience. The results of the tests indicated that the didactical approaches related to students’ metacognitive skills by male and female teachers were significantly different in planning and regulating, but not significant in monitoring and evaluating. Also, the teachers’ didactical approaches related to students’ metacognitive processes did not differ significantly with respect to their academic qualifications and to the years of experience.

It is hoped that the present Special Issue will be proved interesting and useful for the faculties, researchers, teachers, students and in general for all those working in the area of Mathematics Education or being interested on the subject. As the Guest Editors of the Special Issue, we are grateful to the authors of the papers for their quality contributions, to the reviewers for their valuable comments towards the improvement of the submitted works, and to the administrative staff of the MDPI publications for the support to complete this project. Special thanks are due to the Managing Editor of the journal *Ms. Esther Liu* for her excellent collaboration and kind assistance.

## Data Availability

Not applicable.
